# A Sudden and Fatal Case of Purpura

**Published:** 1850-06

**Authors:** Napoleon B. Anderson

**Affiliations:** Louisville, Ky.


Art. II.—*A Sudden and Fatal Case of Purpura.* By Napoleon B. Anderson, M. D., of Louisville, Ky.

The case was that of a boy, Calmore Brown, æt. 9 years, born and raised in this city; free from predisposition to hereditary diseases. He had, previous to the present attack, been a robust, healthy lad; complexion fair; countenance expressive and intelligent; he had symmetry of body, with large and well developed chest.

On February 2d, he commenced expectorating a thick, tough, white, saline sputa, of which no notice was taken, as in other respects he was entirely well. On the 11th, he was attacked with a slight chill, which appeared to centre in the spine. This soon passed off, followed by a high fever; flushed face; pulse one hundred and forty; respiration quick, laborious and hurried, with natural sounds of both lungs; tongue coated, and red at edges and tip; fauces swollen, and of a dark color; the coats of the eyes natural, pupils dilated, and sensible to light. He complained of no pain, with the exception of an uneasiness over the orbits; bowels costive. Ordered hydrarg.

proto-chloride, grs. x., pulv. rhei, grs. iv., jalap. grs. v., made into two powders—one immediately administered, and the second in two hours, followed by oil ricini.

Feb. 12th, 8. A. M.—He has had three free evacuations from the bowels, of a dark, frothy character,—pain over the ileo-cecal valve; urine red, and depositing a brick dust sediment; fever abated; pulse eighty and feeble; tongue darker; pupils contracted and insensible to light; cold perspiration on arms and head; deathly pallor; delirious at times, but sane at intervals; movement of any of the extremities gave excruciating pain; the face, arms, hands, legs and feet were covered with purpuric patches, varying in size from a split pea to that of a dollar. Ordered warm mustard bath, with a desert-spoonful of the following mixture every two hours, in addition to warm brandy toddy and rich chicken soup:

℞. Mucilaginous acacia, ℥iij.,
Carb. ammonia, ʒi.,
Sulph. quinia, grs. xx.,
Aromatic sulphuric acid, q. s.

Misce.

The mouth and tongue were cleansed with fresh lime juice.

12th. 5, P. M.—Delerious, incessantly calling the name of his school teachers, and in trying to fight his play-fellows. Pulse, seventy, and becoming more feeble—pupils still contracted—tonsils same—tongue red at edges, and the surface covered with innumerable red elevated papilla—sordes on the teeth—cold perspiration—hands, arms, feet, and legs cold—cough for the first time appears—expectoration thick and dark—slight congestion of the lungs—the hemorrhagic spots fast increasing in size and number—legs and arms extremely painful—the uneasiness over the cecal valve increasing. Ordered same treatment—repetition of the bath; warm turpentine over the cecum—enema of tepid flaxseed tea ℥ss., oil terebinthina ℨss., tinct. opii. gtts. xxx, in two equal parts, to be administered half hour apart.

He died at 12 o'clock same night.

13th. 9, A. M.—Post mortem objected to. Whole surface of the body ecchymosed, with exudations of bloody mucous from the mouth and nostrils.

Remarks.—This case presents itself to my mind as one of genuine *purpura* or *purples*, which, incorrectly, have been ranked among cutaneous diseases. Its external phenomena are such as to show conclusively that it is a hemorrhage; as pressure upon the spots does not efface the color, nor render it fainter, as it does that of inflammation of the skin. The hemorrhage takes the form of red or purple spots, when the quantity of blood extravasated in the same place is only a drop—and there is scarcely ever any prominence of the purple stigmata—they are livid blotches in appearance exactly resembling bruises—in fact, the anatomical condition of bruises and purpura is the same, changing from red to a greenish yellow—both the result of ecchymosis.

The profession is divided in opinion as regards the true cause of purpura, which difference has given rise to diversified theories. The author is of opinion that the disease is secondary, so far as it develops itself upon the skin—a result of a diseased state of the veins—a softening and breaking down of the different coatings of the entire nervous system—and that the ecchymosis is the result of an exudation of venous blood, with its extravasation into the whole cellular tissue, through the softened structures of the veins. Ecchymosis is seen on the mucous surface of the mouth, the throat, the stomach, the intestines, on the pericardium, pleura, and peritoneal investment of the abdominal organs, in the muscles, on the membranes of the brain, and even on the sheaths of the nerves; and with large extravasations of blood in the most vital organs of the body. Is not purpura, therefore, a disease of the structure of the veins or arteries, or of both, with a contaminating quality existing in the blood, tending in the onset to disorganise their structure? I am of opinion, that taken as a whole, purpura is the result. This, howevęr, is a mere hypothesis, an opinion respectfully expressed.

